# Use of *Shigella flexneri* to Study Autophagy-Cytoskeleton Interactions

**DOI:** 10.3791/51601

**Published:** 2014-09-09

**Authors:** Maria J. Mazon Moya, Emma Colucci-Guyon, Serge Mostowy

**Affiliations:** ^1^Section of Microbiology, MRC Centre for Molecular Bacteriology and Infection, Imperial College London; ^2^Département de Biologie du Développement et des Cellules Souches, Institut Pasteur, Unité Macrophages et Développement de l'Immunité

**Keywords:** Infection, Issue 91, ATG8/LC3, autophagy, cytoskeleton, HeLa cells, p62, septin, *Shigella*, zebrafish

## Abstract

*Shigella flexneri* is an intracellular pathogen that can escape from phagosomes to reach the cytosol, and polymerize the host actin cytoskeleton to promote its motility and dissemination. New work has shown that proteins involved in actin-based motility are also linked to autophagy, an intracellular degradation process crucial for cell autonomous immunity. Strikingly, host cells may prevent actin-based motility of *S. flexneri* by compartmentalizing bacteria inside ‘septin cages’ and targeting them to autophagy. These observations indicate that a more complete understanding of septins, a family of filamentous GTP-binding proteins, will provide new insights into the process of autophagy. This report describes protocols to monitor autophagy-cytoskeleton interactions caused by *S. flexneri in vitro* using tissue culture cells and *in vivo* using zebrafish larvae. These protocols enable investigation of intracellular mechanisms that control bacterial dissemination at the molecular, cellular, and whole organism level.

**Figure:**
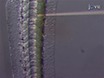


## Introduction

*Shigella flexneri*, a Gram-negative invasive enteropathogenic bacterium, can escape from phagosomes to the cytosol, and polymerize the host actin cytoskeleton to evade cytosolic immune responses and promote intra- and intercellular movement^1,2^. Despite the understanding of actin-based motility *in vitro*^3,4^, the mechanisms restricting bacterial dissemination *in vivo* have not been fully defined. This is critical for a more complete understanding of innate immunity and host defense.

Septins, a highly conserved family of proteins among metazoans, are guanosine triphosphate (GTP)-binding proteins that assemble into hetero-oligomeric complexes and form nonpolar filaments that associate with cellular membranes and the cytoskeleton^5,6^. Recent work has discovered that infected host cells can prevent *Shigella* actin based motility by compartmentalizing bacteria targeted to autophagy inside ‘septin cages’, revealing the first cellular mechanism that counteracts actin based motility^7,8^. A wide open field of investigation now lies in ‘septin biology and infection’. Septin assembly, induced by a variety of pathogens (*e.g.*, *Listeria monocytogenes*^7,9,10^*, Mycobacterium marinum*^7,8^*, Candida albicans*^11^), may emerge as a key issue in host defense^5,12^.

Autophagy, a highly conserved intracellular degradation process, is viewed as a key component of cell-autonomous immunity because of its ability to deliver cytosolic bacteria to the lysosome^13,14^. However, the role of bacterial autophagy *in vivo* to restrict or promote bacterial replication remains poorly understood^15,16^. The zebrafish (*Danio rerio*) has emerged as a vertebrate model for the study of infections because it is optically accessible at the larval stages when the innate immune system is already functional^17,18^. Recent work has characterized the susceptibility of zebrafish larvae to *S. flexneri*, a paradigm for bacterial autophagy^15^, and has used the *Shigella*-zebrafish infection model to study the manipulation of autophagy for antibacterial therapy *in vivo*^19^.

This report provides new tools and assays to study *S. flexneri* interactions with autophagy and the cytoskeleton. In a first step, protocols to monitor autophagy-cytoskeleton interactions are described using *Shigella* infection of the human epithelial cell line HeLa. To assess the role of autophagy-cytoskeleton interactions on the *Shigella *infection process *in vitro*, methods to manipulate autophagy and cytoskeleton components (using siRNA or pharmacological reagents) are provided. New work has shown that by using *Shigella* infection of zebrafish larvae, similar assays can be applied to study the cell biology of infection *in vivo*. Protocols to prepare and infect zebrafish larvae are detailed, and to assess the host response to *Shigella *infection* in vivo*, protocols to determine host survival and bacterial burden of infected larvae are provided. Methods to monitor the recruitment of septin and autophagy markers to *Shigella *(using either fixed or living zebrafish larvae) and methods to test the role of these processes *in vivo *[using morpholino oligonucleotides (injected in 1-4 cell stage embryos) or pharmacological reagents (added directly to the zebrafish bath water)] are also discussed. This program of work is expected to provide insights into the mechanisms required for the control of infection by cytosolic host responses.

## Protocol

### 1. Monitoring Autophagy and the Cytoskeleton *In Vitro* Using Tissue Culture Cells

1. Prepare *S. flexneri*
  1. Plate *S. flexneri* M90T (wild-type) from -80 °C glycerol stock onto a Congo Red tryptic casein soy (TCS) agar plate. Incubate overnight at 37 °C. The same plate can be used for several experiments.
  2. Pick an individual colony and grow in 8 ml TCS media in a shaker overnight at 37 °C. NOTE: Congo-red binding indicates that the virulence plasmid has been retained.
  3. To subculture bacteria for exponential growth, inoculate fresh TCS with the overnight bacterial culture at 1/80 dilution and grow in a shaker at 37 °C to OD_600 _= 0.3-0.6.
  4. Spin the bacterial subculture at 1,000 x g for 5 min. Wash the pellet with MEM and centrifuge at 1,000 x g for 5 min. Reconstitute the pellet in MEM to OD_600 _= 0.3-0.6.
2. Prepare HeLa Cells for Infection
  1. Grow HeLa cells in ‘complete medium’, *i.e*., MEM plus L-alanyl-L-glutamine supplemented with 1 mM sodium pyruvate, 0.1 mM nonessential amino acid solution, and 10% fetal calf serum.
  2. Plate 1-1.5 x 10^5^ cells in 6-well plates 24 or 48 hr before the experiment begins. Plate on glass coverslips in 6-well plates for microscopy, or plate on 35 mm glass bottom dishes to prepare for live imaging. NOTE: To follow autophagy (*e.g.*, ATG8/LC3+ve autophagosomes) and cytoskeleton (*e.g*., actin tails, septin cages) dynamics in real-time during *Shigella* infection using live imaging, tissue culture cells can be transiently transfected using GFP-, RFP- or CFP-tagged constructs (see Discussion).
3. Infection
  1. Infect cells with 100:1 multiplicity of infection (MOI) of *Shigella* (OD_600_ = 0.3 - 0.6) diluted in MEM; directly add to HeLa cells plated in 6-well plates 24 - 48 hr prior to infection (as described in section 1.2) in 2 ml MEM (serum starved).
  2. To maximize bacterial adherence to host cells, centrifuge bacteria and cells at 700 x g for 10 min at room temperature. After centrifugation, incubate for 30 min at 37 °C, 5% CO_2 _and allow infection to proceed.
  3. Wash infected cells twice with fresh MEM and incubate with gentamicin containing complete medium (50 μg/ml, to eliminate extracellular bacteria) for 1-4 hr depending on the experiment.
4. Fixing and Labeling Infected HeLa Cells for Microscopy
  1. Wash infected cells twice with 1x PBS, and fix for 15min in 4% paraformaldehyde in 1x PBS at room temperature. To remove paraformaldehyde, wash cells 2x with 1x PBS.
  2. Incubate fixed cells in 50 mM ammonium chloride for 10 min at room temperature. Wash once with 1x PBS, and permeabilize cells for 4 min with 0.1% octylphenol ethylene oxide condensate at room temperature. NOTE: Alternatives to octylphenol ethylene oxide condensate for permeabilization, such as saponin or methanol, can be applied for different preservation of cellular structures^20^.
  3. Wash cells in 1x PBS and incubate in wet chamber with primary antibodies against autophagy critical components (*e.g.*, p62/SQSTM1) or the septin cytoskeleton (SEPT2, SEPT6, SEPT7, SEPT9, and SEPT11 are expressed in HeLa cells) for 30 min (at room temperature) to overnight (at 4 ºC).
  4. Wash cells twice with 1x PBS and incubate in wet chamber with secondary antibodies, and label filamentous actin (F-actin) with phalloidin, for 30 min (at room temperature) to overnight (at 4 °C). For staining of host cell nuclei add 4",6-diamidino-2-phenylindole (DAPI).
  5. Wash cells in 1x PBS and mount the glass coverslips on slides using mounting medium.
5. Microscopic Imaging of Infected HeLa cells
  1. To image the infected cells use an epifluorescence or confocal microscope and a 63X or 100X objective to identify DAPI-labeled *Shigella*. NOTE: As shown in **Figures 1A**-**1C**, septin cages can be visualized as ring like structures, ~0.6 µm in diameter, surrounding cytosolic bacteria polymerizing actin and recruiting autophagy markers (*e.g.*, p62 and LC3)^7,8^. By contrast, bacteria polymerizing actin tails will not be compartmentalized by septin cages and will not be targeted to autophagy^7,8^.
  2. Using an epifluorescence or confocal microscope and a 63X or 100X objective, quantify the number of intracellular bacteria per microscopic field. Also quantify the number of bacteria entrapped in septin cages and targeted to autophagy, or polymerizing actin tails and escaping autophagy.
  3. To determine the percentage of bacteria entrapped in septin cages or polymerizing actin tails, take a Z-stack image series of infected cells, process the images and count at least 500-1,000 bacteria per experiment from at least 3 independent experiments.

### 2. Functional Analysis of Autophagy and the Cytoskeleton *In Vitro*

NOTE: Both genetic and pharmacological approaches can be used to perturb autophagy in infected tissue culture cells, and the impact of these treatments on the course of infection can be monitored.

1. siRNA-mediated Silencing
  1. Plate 0.8 x 10^5 ^HeLa cells on glass coverslips in 6-well plates in complete medium.
  2. Transfect the following day using a lipid-based transfection reagent with siRNA against autophagy and/or cytoskeleton markers.
  3. After the desired period of incubation, infect the cells with *Shigella* as described in section 1.3.
  4. Fix and label the cells as described in section 1.4.
2. Pharmacological Manipulation NOTE: The cytoskeleton can be manipulated pharmacologically, *e.g.*, to depolymerize the actin cytoskeleton use cytochalasin D or latrunculin B, to depolymerize microtubules use nocodazole, to block actomyosin activity use blebbistatin, or to disrupt septin assembly use forchlorfeneuron. Autophagy can be stimulated by using rapamycin or blocked by using bafilomycin.
  1. To manipulate the cytoskeleton during *Shigella* infection, first infect the cells with *Shigella* as described in section 1.3 and allow sufficient time for bacteria to enter cells and escape from the phagosome to the cytosol (*e.g*., >1.5 hr post infection).
  2. Dilute the drugs from the stock solution [stock solution suspended in dimethyl sulfoxide (DMSO)] in MEM to a final concentration of 5 M (cytochalasin D, latrunculin B, nocodazole), 20 M (forchlorfeneuron) or 50 M (blebbistatin), and treat cells 30 min at 37 °C. The total amount of drug (volume of the DMSO/drug mixture) added per plate/vial of cells is 1-5 µl (depending on stock solution) per 2 ml of media. Treat cells with a similar dose of DMSO diluted in MEM as a negative control.
  3. Fix and label the cells as described in section 1.4. NOTE: For manipulation of autophagic flux (in infected or noninfected cells), extend drug treatment using rapamycin (20 nM) or bafilomycin (160 nM) from 4 to 12 hr.
3. Western Blot NOTE: Autophagic activity can be quantified by measuring the protein level of autophagy markers such as p62 and LC3.
  1. After the desired period of incubation, collect and lyse the cells for immunoblotting. Run protein extracts on 8, 10, or 14% acrylamide gels.
  2. Autophagic flux, *i.e*. the rate of autophagy, can be analyzed as described in ^21,22^.
4. Microscopic Imaging and Quantification
  1. Using an epifluorescence or confocal microscope and a 63X or 100X objective, the effect of siRNA or drug treatment on *Shigella* infection can be evaluated by quantitative microscopy (*i.e.*, counting of autophagosomes, septin cages and actin tails) as described in section 1.5 and shown in **Figures 2A** and **2B**.

### 3. *In Vivo* Imaging of *S. flexneri* Interactions with Autophagy and the Cytoskeleton

NOTE: The zebrafish model of *Shigella* infection can be used to investigate septin caging and autophagy *in vivo*^19^.

1. Prepare *S. flexneri*
  1. Culture *S. flexneri* as described in section 1.1.
  2. At OD_600_ = 0.3-0.6, spin 8 ml bacterial subculture at 1,000 x g for 10 min. Wash the pellet with 1x PBS and centrifuge at 1,000 x g for 10 min.
  3. Resuspend the pellet in 80 µl of 0.1% phenol red 1x PBS to obtain ~2,000 bacteria/nl. Keep the bacterial preparation on ice to slow down growth. NOTE: Adding phenol red will help to visualize the inoculum when injecting into the larvae.
2. Prepare Zebrafish Larvae for Injection NOTE: Zebrafish are laid as eggs and are identified as embryos until 72 hr post fertilization, when they are called larvae.
  1. Breed adult zebrafish as described in Westerfield^23^ by placing 4 males and 8 females (usually a 2:1 ratio) into a separate fish tank with the bottom covered with marbles (that will prevent adults from eating the spawned eggs). Alternatively, place egg collection baskets inside the breeding tanks the night before. NOTE: Eggs are fertilized ~30 min after the lights go on in the zebrafish facility^23^, and should be collected as soon as possible to prevent mold growth. Egg collection baskets serve to collect the eggs so they can be easily harvested and also protect the eggs from adults.
  2. Collect the embryos and clean them by washing in embryo media (E2) with 0.003% bleach for 10 min. Remove E2 with bleach, wash embryos 5x in E2 medium, and grow the embryos in 10 cm Petri dish (100 embryos/50 ml E2 medium) at 28 °C.
  3. If embryos or larvae will be used for microscopy studies, at 24 hr post fertilization add 0.003% N-phenylthiourea to the E2 medium to prevent melanization. Keep the embryos at 28 ºC for normal development. NOTE: Zebrafish larvae are ready for infection at 72 hr post fertilization.
  4. For infection and microscopy procedures, anesthetize zebrafish larvae in 200 g/ml tricaine in E2.
3. Preparation of Zebrafish Larvae for Intravenous and Local Infection NOTE: To assess zebrafish survival during *Shigella* infection, perform caudal intravenous injections. To visualize the recruitment of septin and autophagy markers to *Shigella*, perform infection at localized sites such as the tail muscle.
  1. For a caudal intravenous injection, position the anesthetized larvae laterally with the dorsal side facing the needle. As shown in **Figure 3A**, place the needle tip close (posterior) to the urogenital opening, aiming for the caudal vein, and pierce the skin and deliver the desired bacterial dose (injection volume 1-5 nl). NOTE: Intravenous infection is challenging to perform and will take several weeks of training to get comfortable with this procedure. Injecting phenol red (without bacteria) for training will help to assess the injection site properly. NOTE: In the case of *Shigella*, dose dependent experiments have shown that a low dose infection (<1,000 CFU) is cleared within 48 hr, and a high dose infection (>4,000 CFU) leads to host mortality within 48 hr^19^.
  2. For a tail muscle infection, position the anesthetized larvae as described in section 3.3.1. As shown in **Figure 3A**, place the needle carefully over muscle somites (*i.e.*, segments of skeletal muscle) and inject a small volume (*i.e*., 1 nl) of bacterial preparation.
4. Intravenous Injection of Bacteria in Larvae
  1. Pull borosilicate glass microcapillaries as described in ^24^.
  2. Connect needle to the holder of the three-dimensional coarse manual manipulator and break the needle tip with fine tweezers.
  3. To load the needle, place a drop of bacterial culture onto a coverslip. Switch on the microinjector and gas cylinder, slightly submerge the needle tip into the drop, and fill up the needle with the desired amount of bacterial preparation.
  4. To calibrate the injection volume, place a drop of mineral oil on a cover slip and inject the bacterial preparation. Measure the diameter of the drop using a micrometer and calculate the injected volume [V= (4/3)πr^3^]. NOTE: Using injection settings of 40 psi and 50 msec with a bacterial preparation as described in section 3.1 will give ~2,000 CFU/nl.
  5. Prepare the injection plate using a plastic mold as described in Westerfield^23^.
  6. Transfer the larvae to the injection plate and line them up using a fine paintbrush. Orient and inject the larvae as described in section 3.3.1.
  7. For assessment of zebrafish survival, transfer infected larvae individually in 24-well plates in 1 ml of E2/well and incubate at 28 °C. Monitor the infected larvae daily for the next 2-5 days and plot survival over time (**Figure 3B**).
5. Plating Zebrafish Larvae for Bacterial Quantification NOTE: Work under a sterile hood to avoid contamination.
  1. To evaluate the number of bacteria injected into the fish (at time 0 hr post infection) and for bacterial quantification at desired time points, sacrifice zebrafish larvae with an overdose of tricaine (200-500 mg/L). Place individual larvae in 1.5 ml polypropylene microcentrifuge tube with 200 µl of 0.1% octylphenol ethylene oxide condensate 1x PBS and mechanically homogenize using a pestle. NOTE: To confirm the bacterial load in the injection volume, pump an equal dose into a sterile 1x PBS drop and plate it out.
  2. Make a serial dilution of the zebrafish larvae homogenates in sterile water and plate onto lysogeny broth (LB) agar plates. Larvae can be plated alternatively on Congo Red TCS plates to discriminate between *Shigella* having maintained the virulent plasmid or not. NOTE: Plate 3 or more noninfected fish as a control to check the status of the zebrafish larvae used for the infection.
  3. After overnight incubation of plates at 37 °C, count bacterial colonies. As shown in **Figure 3C**, represent using a log scale. NOTE: Bacterial load during infection of zebrafish larvae can also be visualized using fluorescently labeled *Shigella *and microscopic imaging as described in section 3.7 or 3.8 (**Figure 3D**).
6. Zebrafish Larvae Immunostaining
  1. At desired time points, sacrifice the larvae using an overdose of tricaine. Collect the fish in 1.5 ml polypropylene microcentrifuge tubes (10 to 20 larvae/tube).
  2. Fix the larvae using 4% paraformaldehyde with 0.4% octylphenol ethylene oxide condensate in 1x PBS and incubate in an orbital agitator (to avoid larval clustering) for 2 hr (at room temperature) or overnight (at 4 °C). NOTE: Electron microscopy can be used for ultrastructural analyses of infected zebrafish larvae. In this case, anesthetized embryos should be fixed and processed according to Mostowy *et al*^19^.
  3. Wash 3x in 1x PBS 0.4% octylphenol ethylene oxide condensate for 5 min, then block in blocking solution (10% fetal calf serum, 1% DMSO, 0.1% polyoxyethylenesorbitan monolaurate in 1x PBS) for 1 hr at room temperature.
  4. Dilute the primary antibody in blocking solution. Add larvae to diluted primary antibody and incubate overnight at 4 °C in an orbital agitator. Use primary antibodies as described above in section 1.4.3.
  5. Wash the larvae 4x for 15 min in 0.1% polyoxyethylenesorbitan monolaurate 1x PBS at room temperature.
  6. Dilute the secondary antibody in blocking solution. Add larvae to diluted secondary antibody and incubate overnight at 4 °C in an orbital agitator. Use the same secondary antibodies and phalloidin as described in section 1.4.4.
  7. Wash 4x for 15 min in 0.1% polyoxyethylenesorbitan monolaurate 1x PBS at room temperature. For staining of host cell nuclei add DAPI (150 nM final concentration) during the first of these 15 min washes.
  8. For preservation of fluorescently-labeled larvae, incubate them progressively in a glycerol gradient of 15, 30, 60, and 80% diluted in 1x PBS and 0.1% polyoxyethylenesorbitan monolaurate for 2 hr (at room temperature) to overnight (at 4 °C). NOTE: Stained larvae can be stored for long periods of time (*e.g*., 4 months) in 80% glycerol at 4 °C.
7. Microscopic Imaging of Fixed Zebrafish Larvae
  1. Transfer fixed glycerol embedded larvae to a small drop of 80% glycerol in a 35 mm Petri dish (for stereomicroscopy) or full glass bottom dish (for confocal miscopy).
  2. Take Z-stack image series of infected larvae and process the images as required.
  3. Use an epifluorescence or confocal microscope and a 10X or 20X objective for whole organism imaging. Then use a confocal microscope and a 40X, 63X, or 100X objective to visualize individual cells and the recruitment of autophagy and cytoskeleton markers to individual bacteria *in vivo*^19^ **(Figures 4A **and **4B**). NOTE: Infect fish in the tail muscle and mount in glycerol flat along the bottom of the glass bottom dish to enable easy focus.
8. Live Microscopic Imaging of Infected Zebrafish Larvae NOTE: Larvae are optically accessible, thus *in vivo* autophagosomes can be visualized using the GFP-Lc3 zebrafish transgenic line^25^. Infect zebrafish larvae as described in section 3.3 and mount as described in this section.
  1. Prepare low-melting 1% agarose (LMA) in E2 and allow to cool to 35-37 °C to avoid larvae damage/killing. Distribute drops of LMA in a 35 mm Petri dish (for stereomicroscopy) or full glass bottom dish (for confocal microscopy).
  2. Transfer anesthetized zebrafish larvae individually (with as little water as possible) to the LMA drops. Orient larvae to the desired position using a paintbrush and wait for the agarose to solidify.
  3. Cover the whole dish surface with LMA, and overlay with E2 containing 200 µg/ml tricaine to avoid the preparation from drying out and to allow fish to exchange oxygen from the water.
  4. Use an epifluorescence or confocal microscope and a 10X or 20X objective for imaging the entire zebrafish larvae. Use a confocal microscope and a 40X, 63X, or 100X objective to visualize bacterial authopagosomes (*i.e.*, GFP-Lc3+ve vacuoles surrounding *Shigella*) *in vivo*.
  5. Take a Z-stack of infected larvae over time (*e.g*., every 2 min over several hr) to visualize autophagosomes, and their dynamics, in real time.

### 4. Functional Analysis of Autophagy and the Cytoskeleton *In Vivo*

NOTE: The impact of pharmacological and genetic perturbations of autophagy on the course of infection can be monitored at the whole-animal level, and at the level of the single cell.

1. Autophagy Manipulation by Morpholino Injection
  1. Reconstitute morpholino oligonucleotides in sterile water to a stock solution of 1 mM by warming at 65 °C for 10 min. Store at room temperature. NOTE: morpholino oligonucleotide injections must be performed in 1-4 cell stage embryos.
  2. Prepare morpholino oligonucleotide working solution with sterile 0.1% phenol red in Dulbecco’s phosphate buffered saline. Load the needle as described in section 3.4.2., morpholino oligonucleotide injection volume can be calibrated as described in section 3.4.3. NOTE: Phenol red will help to visualize the volume injected.
  3. Prepare an embryo-positioning chamber (*i.e*., a microscope slide glued with cyanoacrylate on a 10 cm Petri dish lid with edges facing the needle partially removed). Transfer 1-4 cell stage embryos to the chamber with a small amount of water and align them with a fine paintbrush.
  4. Penetrate the chorion and the yolk smoothly. Once inside, press the pedal to inject the desired volume of morpholino oligonucleotide solution. NOTE: Minimize the volume of injection to 0.5 - 2 nl; volumes higher than 5 nl may cause developmental defects and increase egg mortality.
  5. After microinjection, clean embryos (by bleaching as described in section 3.2) and incubate them in a petri dish with E2 at 28 °C.
  6. Infect 72 hr post fertilization control (*i.e.*, zebrafish larvae injected with control morpholino oligonucleotide) or p62 morphants (*i.e*., zebrafish larvae injected with p62 morpholino oligonucleotide) with *Shigella* as described in section 3.4. Assess survival and bacterial burden for the next 2-5 days as described in section 3.5. Image fixed zebrafish larvae as described in section 3.7 and highlighted in **Figure 4C**, or image living zebrafish larvae as described in section 3.8. NOTE: The effective morpholino oligonucleotide dose can be assessed based on its efficiency to inhibit transcript splicing or protein translation (see Discussion).

## Representative Results

Upon infection of tissue culture cells *in vitro*, *S. flexneri* can escape from the phagosome and invade the cytosol. In the cytosol, host cells can prevent the actin-based motility of *Shigella* by compartmentalizing bacteria inside septin cages (**Figure 1A**). Bacteria entrapped by septin cages can also be labeled by autophagy markers p62 (**Figure 1B**) and LC3 (**Figure 1C**). These observations highlight a novel mechanism of host defense that restricts dissemination of invasive pathogens, and also reveal new links between autophagy and the cytoskeleton. Strikingly, the depletion of autophagy markers significantly reduces septin caging of bacteria (**Figure 2A**), and work has also shown that the depletion of septin caging significantly reduces recruitment of autophagy markers^8^. Thus, at least in the case of* Shigella*, septin cage assembly and autophagosome formation can be viewed as interdependent processes. Other cellular requirements for compartmentalization of *Shigella* by septin cages include actin polymerization and actomyosin activity (**Figure 2B**).

There is no natural mouse model of shigellosis, and investigation of *Shigella* pathogenesis, septin biology and bacterial autophagy *in vivo *can benefit from a new animal model of infection, the zebrafish larvae^19^. It is possible to infect zebrafish larvae by injecting bacteria in various anatomical sites such as caudal intravenous injections for survival experiments, and tail muscle injections for *in vivo* microscopy (**Figure 3A**). Depending on the dose, *S. flexneri* injected in zebrafish larvae can either be cleared within 48 hr post-infection, or may result in a progressive and ultimately fatal infection (**Figures 3B**-**3D**). *Shigella* virulence factors are expressed at 28 ° C, the optimal growth temperature of zebrafish, and zebrafish infection by *Shigella *is strictly dependent upon its type III secretion system (T3SS)^19^, an essential virulence determinant in human disease. Taken together, these observations indicate that the zebrafish larva represents a valuable new host for *in vivo* analysis of *Shigella* infection.

The optical accessibility of zebrafish larvae enables visualization of septin caging *in vivo* (**Figure 4A**), an achievement that has never before been accomplished using mammalian host models. To complement evidence that septin cages entrap bacteria targeted to autophagy *in vivo*, one can infect transgenic zebrafish larvae expressing GFP-Lc3 and observe autophagy marker recruitment to *Shigella* (**Figure 4B**). For ultrastrucutral analysis of *Shigella* autophagosomes *in vivo*, electron microscopy can be used to clearly show the cytosolic sequestration of bacteria by double membrane vacuoles^19^. Autophagy is viewed as a key component of cell-autonomous immunity and a crucial defense mechanism against intracytosolic bacteria^14-16^. To characterize autophagy function* in vivo*, p62 morpholino-treated zebrafish larvae can be used. Unlike the core autophagy machinery [*e.g*., the 36 autophagy related proteins (ATGs)^26^], p62 is not essential for vertebrate development^27^ and thus zebrafish larvae can develop normally prior to infection. Strikingly, p62-depleted larvae inoculated with *S. flexneri* result in significantly increased mortality and increased bacterial burden^19^. In agreement with *in vitro* work showing that septin cage assembly is interdependent with autophagosome formation^7,8^, septin recruitment to *Shigella* is clearly reduced in p62-depleted larvae (**Figure 4C**). These data demonstrate that zebrafish survival depends on p62-mediated autophagy to control intracellular bacterial infection.


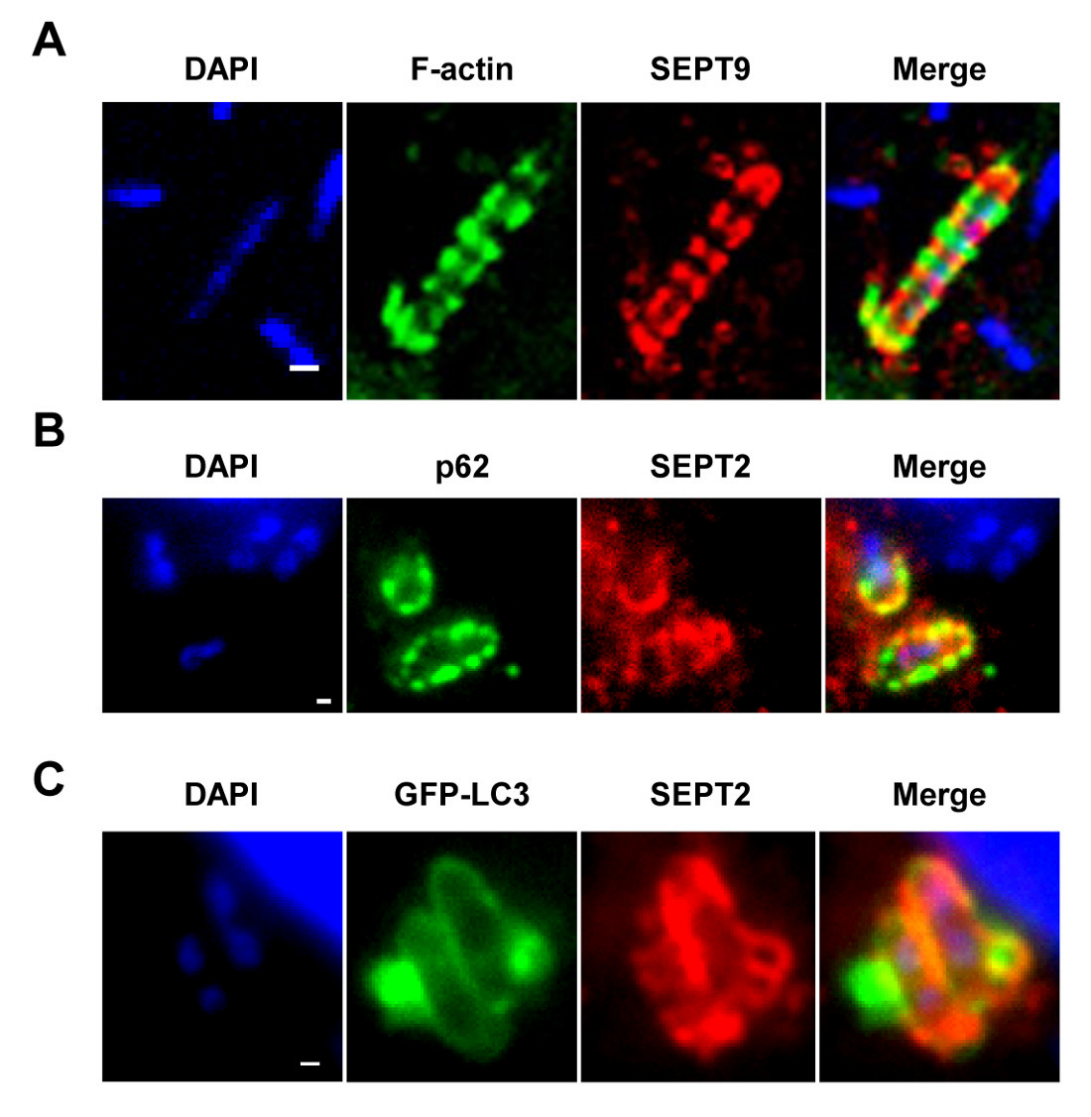
**Figure 1. The septin cage* in vitro*.(A) **HeLa cells were infected with *S. flexneri* for 4 hr 40 min, fixed, labeled with antibodies to SEPT9 and phalloidin, and imaged by confocal microcopy. Scale bar, 1 µm. **(B)** HeLa cells were infected with *S. flexneri* for 4 hr 40 min, fixed, labeled with antibodies to p62 and SEPT2, and imaged by fluorescent light microscopy. Scale bar, 1 µm. **(C)** HeLa cells were transfected with GFP-ATG8/LC3, infected with *S. flexneri* for 4 hr 40 min, fixed, labeled with antibodies to SEPT2, and imaged by fluorescent light microscopy. Scale bar, 1 µm. These figures have been modified from Mostowy *et al*^7^.


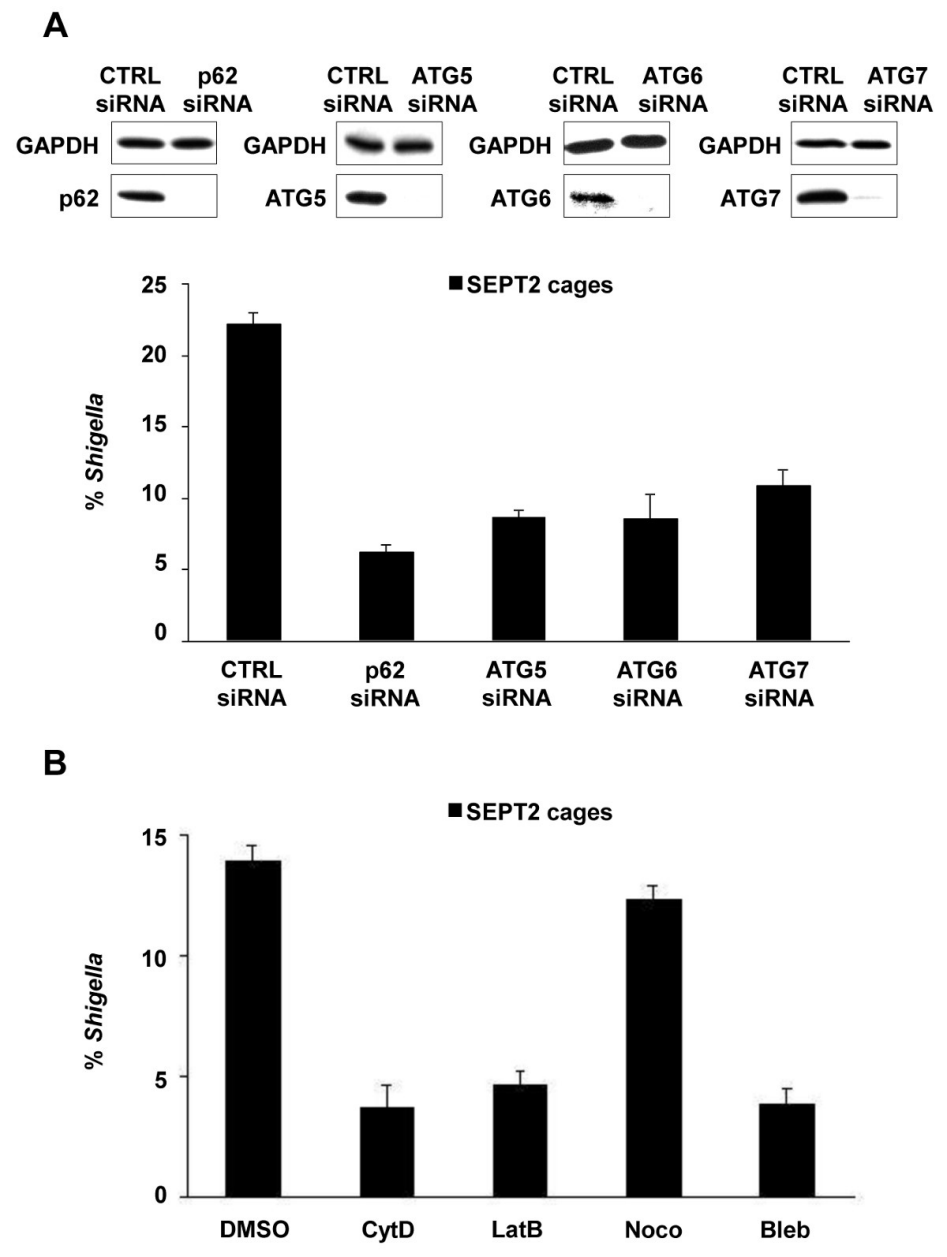
**Figure 2.** **Cellular requirements for *Shigella*-septin cage formation.** **(A)** HeLa cells were treated with control (CTRL), p62, ATG5, ATG6 or ATG7 siRNA. Whole-cell lysates of siRNA-treated cells were immunoblotted for GAPDH, p62, ATG5, ATG6, or ATG7 to show the efficiency of siRNA depletion (top). siRNA-treated cells were infected with *S. flexneri *for 4 hr 40 min, fixed, and labeled for quantitative microscopy. Graphs (bottom) represent the mean % ± SEM of *Shigella *inside SEPT2 cages from n ≥3 experiments per treatment**. (B)** HeLa cells were infected with *S. flexneri*, treated with DMSO, cytochalasin D (CytD), latrunculin B (LatB), nocodazole (Noco), or blebbistatin (Bleb) and after 4 hr 40 min were fixed and labeled for quantitative microscopy. Graphs represent the mean % ± SEM of *Shigella* inside SEPT2 cages from two independent experiments per treatment**. **These figures have been modified from Mostowy *et al*^7^**.**


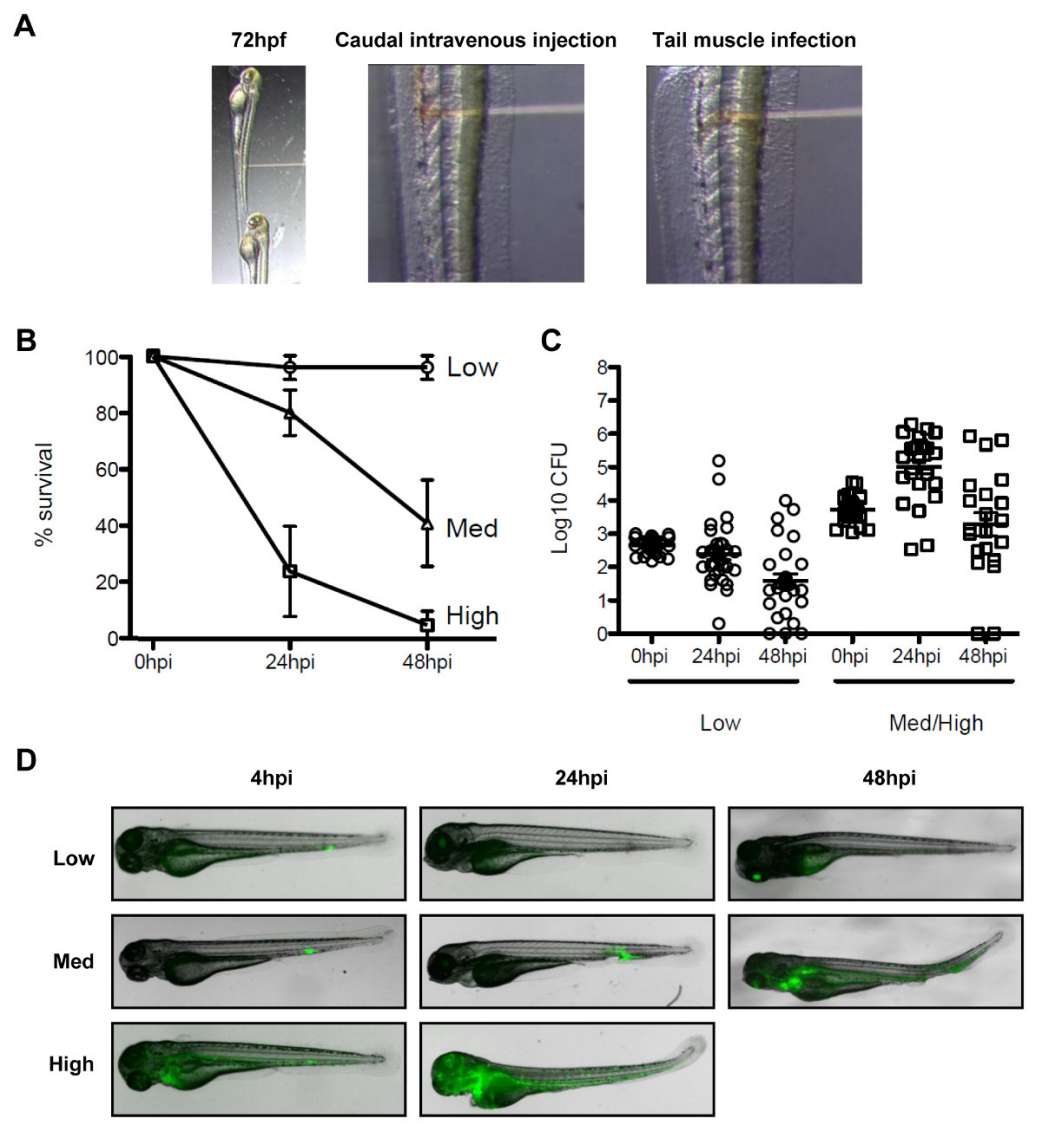
**Figure 3. The zebrafish model of *Shigella* infection. (A)** Images to illustrate orientation of the zebrafish larva under the stereomicroscope. (Left panel) Zebrafish larvae 72 hr post fertilization were positioned laterally in the injection plate with their dorsal side facing the injection needle. (Middle panel) Bloodstream infection was performed by injecting the bacteria (red solution) in the caudal vein, posterior to the urogenital opening. (Right panel) Infection in the tail muscle was performed by injecting the bacteria (red solution) over a somite. **(B)** Survival curves of 72 hr post fertilization larvae injected with various doses of *S. flexneri* and incubated at 28 °C for 48 hr post infection. The effective inoculum was classified as low (<10^3 ^CFU, open circles), medium (~4 x 10^3^ CFU, open triangles) or high (~10^4^ CFU, open squares). Mean % ± SEM (horizontal bars) from n ≥3 experiments per inoculum class. **(C)** Enumeration of live bacteria in homogenates from individual larvae at various times post infection measured by plating onto LB. Note, only larvae having survived the infection are included in enumeration analysis. Mean ± SEM (horizontal bars) also shown. **(D)** Distribution of GFP-*Shigella* determined by live imaging using a fluorescent stereomicroscope at various times post infection using a low, medium, or high dose inoculum (caudal intravenous injections). Overlay of transmission image (grey) and GFP fluorescence (green). **(B)**-**(D)**These figures have been modified from Mostowy *et al*^19^.


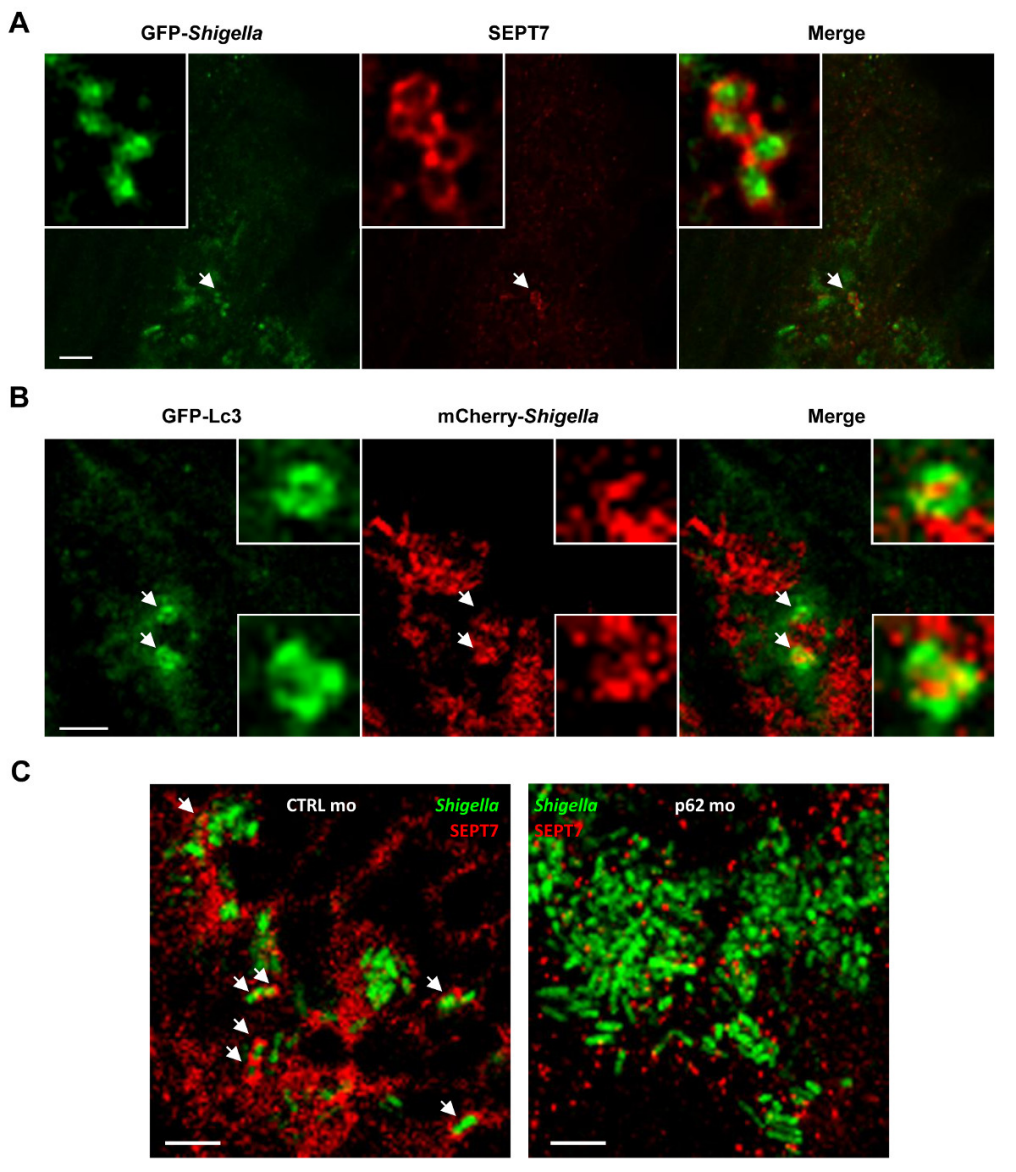
**Figure 4.** **The cell biology of *Shigella* infection *in vivo*.** **(A)** Zebrafish larvae were infected in the tail muscle with GFP-*Shigella* (low dose) for 24 hr, fixed, labeled with antibodies against SEPT7 (red) and to GFP (green), and imaged by confocal microscopy. Scale bar, 5 µm. **(B)** GFP-Lc3 zebrafish larvae were infected with mCherry-*Shigella* (medium dose) for 4 hr, fixed, labeled with antibodies against mCherry (red) and to GFP (green), and imaged by confocal microscopy. Scale bar, 1.5 µm. **(C)** Zebrafish larvae treated with either control (CTRL; left image) or p62 (right image) morpholinos were infected with GFP-*Shigella* for 4 hr (medium dose), fixed, labeled with antibodies against SEPT7 (red) and to GFP (green), and imaged by confocal microscopy. Arrows highlight some examples of *Shigella* entrapped in septin cages (CTRL) or not (p62 depleted) a 4 hr post infection. Scale bar, 5 µm. These figures have been modified from Mostowy *et al*^19^.

## Discussion

When monitoring autophagy and the cytoskeleton *in vitro* using tissue culture cells, the protocols described in sections 1 and 2 can be applied to a wide variety of tissue culture cell types. Moreover, to follow autophagy (*e.g.*, ATG8/LC3+ve autophagosomes) and cytoskeleton (*e.g*., actin tails, septin cages) dynamics in real-time during *Shigella* infection using live imaging, tissue culture cells can be transiently transfected using GFP-, RFP- or CFP-tagged constructs as previously described^7,8^. To increase the percentage of cells infected by *Shigella* (*i.e*., generally desirable for real-time analysis considering that *Shigella* can invade 5-30% of HeLa cells at 100:1 MOI), directly add 400 µl of *Shigella* (OD_600_ = 0.3-0.6) to cells in 2 ml MEM (serum-starved) and wait at least 1.5 hr post infection for sufficient bacterial entry, escape from the phagosome, replication, autophagy recognition and septin caging. Alternatively, one may use the *Shigella *M90T AfaI strain that expresses the adhesin AfaE and have much higher invasion abilities in epithelial cells compared to the M90T strain^28^. Of note, the M90T AfaI strain has not yet been tested *in vivo* using zebrafish. Plates of *Shigella* colonies can be kept at 4 °C for 2-3 days and used for several experiments. However, over time, colonies of *Shigella* that have lost the virulence plasmid can also absorb the Congo Red and appear to have retained their virulence plasmid. For this reason we recommend to use fresh bacterial stocks when possible.

When monitoring the cell biology of infection *in vivo*, protocols described in sections 3 and 4 use wildtype AB line zebrafish. To monitor *Shigella*-leukocyte interactions, transgenic zebrafish lines can be used, *e.g.*, *mpx*:GFP or *lyz*:DsRed to visualize neutrophils^19,29,30^ or *mpeg1*:mcherry to visualize macrophages^19,31^. To visualize autophagy *in vivo*, the GFP-Lc3 zebrafish transgenic line^19,24^ can be used as described in section 3.8.

To perturb autophagy *in vivo*, the effective morpholino oligonucleotide dose has to be assessed experimentally based on its efficiency to inhibit transcript splicing or protein translation. It is advisable to perform a titration experiment and to confirm the depletion by RT-PCR (for splice morpholino oligonucleotide) or by SDS-PAGE (for translational morpholino oligonucleotide)^32^. RNA isolation from zebrafish embryos or larvae can be performed using guanidinium thiocyanate-phenol-chloroform extraction. To extract protein from zebrafish larvae (8 to 15 larvae/tube), mechanically homogenize using a pestle in 200 µl lysis buffer (1 M Tris, 5 M NaCl, 0.5 M EDTA, 0.01% octylphenol ethylene oxide condensate, and protease inhibitor). Centrifuge tubes at 19,000 x g at 4 ° C for 15 min and transfer the supernatant to a new tube. Add Laemmli buffer and heat the sample at 95 ° C for 15 min. Lysates can be stored at -80 ° C until needed, and can be evaluated by Western blotting as described in section 2.3.

The zebrafish is an excellent model for *in vivo *drug application. Analysis using morpholino oligonucleotides can be complemented with established drugs to manipulate autophagy (*e.g.*, rapamycin and bafilomycin). Uninfected and/or infected larvae can be treated with rapamycin (1.5 µM) or bafilomycin (80 nM) diluted in E2 and autophagic flux can be evaluated by Western blotting as described in ^25,33^. The consequence of autophagy manipulation on the outcome of the infection and survival of the infected larvae can be evaluated as described in section 3.5.

In addition to studying host cell determinants, *in vitro* and* in vivo* protocols can be applied to assess bacterial determinants required for autophagy recognition, using bacterial mutant strains that are differentially recognized by autophagy, *e.g*., *ShigellaΔicsA *(the *Shigella *protein IcsA recruits N-WASP and then Arp2/3 for actin tail and septin cage formation; in its absence there can be no actin tails, no septin cages) and *ShigellaΔicsB *(*Shigella* avoids the autophagic response via the bacterial effector protein IcsB, which prevents the recruitment of autophagy machinery to *IcsA; *in its absence there can be more septin cages, more autophagy)^7,8^.

*Shigella* is not a natural pathogen of zebrafish and grows optimally at 37 °C. However, work has shown that virulence factors required for *Shigella* invasion, escape from the phagocytic vacuole and replication in the cytosol can be expressed and are functional in zebrafish larvae at 28 °C^19^. 28 °C is the most commonly used temperature for zebrafish rearing and standard temperature to ensure normal zebrafish development^23^. Strikingly, the major pathogenic events that lead to shigellosis in humans (*i.e.*, macrophage cell death, invasion and multiplication within epithelial cells, cell-to-cell spread, inflammatory destruction of the host epithelium) are faithfully reproduced in the zebrafish model of *Shigella* infection^19^.

Autophagy and cytoskeleton genes are ubiquitously expressed and have a wide range of biological functions. Mouse studies have shown that knockout of essential autophagy^26^ or septin genes^5^ are embryonic lethal, and it is likely that some of these genes will also be essential for zebrafish development (although this problem may be reduced by the fact that zebrafish have multiple paralogous genes^33^). If so, there are several alternatives to overcome this issue, including (i) the use of pharmacological reagents to regulate autophagy and the cytoskeleton, (ii) morpholinos can be titrated down, (iii) knockout of genes can be designed for only specific cell types, and/or (iv) genes involved in autophagic recognition that are not essential for animal development (*e.g*., p62) may be targeted.

While the zebrafish is an ideal model system to investigate autophagy and the cytoskeleton during *Shigella* infection, molecular tools are currently lacking. The field needs to generate new tools and drive cell specific expression of the proteins of interest. To knock down expression of autophagy/cytoskeleton genes, new morpholino sequences are required, and novel methods for genome engineering (*e.g.*, TALEN, CRISPR/Cas9) can also be used. In the meantime, several tools previously generated for human or mouse studies may equally work for zebrafish.

The intracellular bacteria *S. flexneri *has emerged as an exceptional model organism to address key issues in biology, including the ability of bacteria to be recognized by the immune system^1,2^. The host cell employs septins to restrict the motility of *S. flexneri* and target them to autophagy, a critical component of cell autonomous immunity^7,8^. These observations suggest a new molecular framework to study autophagy and its ability to degrade cytosolic bacteria. A major issue is now to fully decipher the underlying molecular and cellular events, and to validate these events analyzed *in vitro* during bacterial infection *in vivo* using relevant animal models. To this end, the zebrafish has been established as a valuable new host for the analysis of *S. flexneri* infection^19^. Interactions between bacteria and host cells can be imaged at high resolution, and the zebrafish model should prove useful for understanding the cell biology of *Shigella* infection *in vivo*. Zebrafish larvae can be used to investigate the role of bacterial autophagy in host defense, and work has shown that that the perturbation of autophagy can adversely affect host survival in response to *Shigella* infection^19^.

The observations generated from study of *Shigella*, septin caging and autophagy* in vitro *using tissue culture cells and *in vivo* using zebrafish larvae might provide fundamental advances in understanding host defense. They could also suggest the development of new strategies aimed at combating infectious diseases.

A critical aim of this report is to make sense of the molecular and cellular events analyzed *in vitro* (*i.e.*, autophagy, actin tails, septin caging) during bacterial infection *in vivo* in the context of an entire organism, using zebrafish larvae. If not familiar with zebrafish biology and handling, one may refer to in depth protocols for proper zebrafish husbandry^23^ and *in vivo *analysis of zebrafish infection^19,35^.

## Disclosures

The authors declare that they have no competing financial interests.
